# Artificial Intelligence in the Diagnosis of Neurological Diseases Using Biomechanical and Gait Analysis Data: A Scopus-Based Bibliometric Analysis

**DOI:** 10.3390/neurolint17030045

**Published:** 2025-03-20

**Authors:** Aikaterini A. Tsiara, Spyridon Plakias, Christos Kokkotis, Aikaterini Veneri, Minas A. Mina, Anna Tsiakiri, Sofia Kitmeridou, Foteini Christidi, Evangelos Gourgoulis, Triantafylos Doskas, Antonia Kaltsatou, Konstantinos Tsamakis, Dimitrios Kazis, Dimitrios Tsiptsios

**Affiliations:** 1 Third Department of Neurology, Aristotle University of Thessaloniki, 541 24 Thessaloniki, Greece; katetsiaa@gmail.com (A.A.T.); vaggeg2000@gmail.com (E.G.);; 2 Department of Physical Education and Sport Science, University of Thessaly, 421 00 Trikala, Greece; spyros_plakias@yahoo.gr (S.P.); ksveneri@gmail.com (A.V.); akaltsat@gmail.com (A.K.); 3 Department of Physical Education and Sport Science, Democritus University of Thrace, 691 00 Komotini, Greece; ckokkoti@affil.duth.gr; 4 Department of Sport, Outdoor and Exercise Science, School of Human Sciences & Human Sciences Research Centre, University of Derby, Kedleston Road, Derby DE22 1GB, UK; m.mina@derby.ac.uk; 5 Neurology Department, Democritus University of Thrace, 681 00 Alexandroupoli, Greece; anniw_3@hotmail.com (A.T.); s.kitmer@gmail.com (S.K.); christidi.f.a@gmail.com (F.C.); 6 Athens Naval Hospital, 115 21, Athens, Greece; doskastr@gmail.com; 7 South London and Maudsley NHS Foundation Trust, Bethlem Royal Hospital, Monks Orchard Road, Beckenham, London BR3 3BX, UK

**Keywords:** machine learning, deep learning, computer vision, diagnosing, severity, rehabilitation, occupational therapy, neurology

## Abstract

Neurological diseases are increasingly diverse and prevalent, presenting significant challenges for their timely and accurate diagnosis. The aim of the present study is to conduct a bibliometric analysis and literature review in the field of neurology to explore advancements in the application of artificial intelligence (AI) techniques, including machine learning (ML) and deep learning (DL). Using VOSviewer software (version 1.6.20.0) and documents retrieved from the Scopus database, the analysis included 113 articles published between 1 January 2018 and 31 December 2024. Key journals, authors, and research collaborations were identified, highlighting major contributions to the field. Science mapping investigated areas of research focus, such as biomechanical data and gait analysis including AI methodologies for neurological disease diagnosis. Co-occurrence analysis of author keywords allowed for the identification of four major themes: (a) machine learning and gait analysis; (b) sensors and wearable health technologies; (c) cognitive disorders; and (d) neurological disorders and motion recognition technologies. The bibliometric insights demonstrate a growing but relatively limited collaborative interest in this domain, with only a few highly cited authors, documents, and journals driving the research. Meanwhile, the literature review highlights the current methodologies and advancements in this field. This study offers a foundation for future research and provides researchers, clinicians, and occupational therapists with an in-depth understanding of AI’s potentially transformative role in neurology.

## 1. Introduction

Neurological disorders are the leading cause of sickness and disability globally, presenting significant challenges for early diagnosis and effective management [1]. From 1990 to 2021, there has been an 18% increase in disability-adjusted life years (DALYs) associated with neurological diseases, which reflects the rising impact of neurological disease on global health [2]. While age-standardized DALY rates have decreased due to advancements in care, the absolute number of people affected continues to grow due to demographic shifts and longer lifespans [3]. Importantly, there is a neurological health loss of over 80% in low- and middle-income nations, where access to therapy and health facilities remains limited [2,4]. In 2021, over three billion people worldwide had a neurological disorder [2], including a wide range of diseases that affect the brain, spinal cord, and nervous system, with symptoms ranging from motor and sensory impairments to cognitive dysfunction, along with Alzheimer’s disease, Parkinson’s disease, multiple sclerosis, epilepsy, and stroke, as well as neurological damage caused by infections, tumors, and trauma [2].

The early detection and classification of neurological disorders are critical for mitigating disease progression and improving patient outcomes. However, the complex and dynamic nature of the nervous system poses significant diagnostic challenges. Traditional tools such as electroencephalograms (EEGs), magnetic resonance imaging (MRI), and computed tomography (CT) scans provide valuable insights, but often fall short in precision and scalability, particularly for early detection [5,6,7,8,9]. In this context, artificial intelligence (AI) techniques, including machine learning (ML) and deep learning (DL), have emerged as transformative solutions. By leveraging computational power and data-driven algorithms, AI enables the advanced analysis of neuroimaging data and delivers quantitative assessments that surpass conventional methods [10,11,12,13]. The integration of neuroscience and AI has strengthened this relationship, facilitating practical applications for detecting and classifying various neurological conditions, particularly in analyzing large datasets like biomechanical data [14,15].

Advances in smart wearable technology, multi-modal sensors, and AI-driven platforms have made it possible to assess gait and balance with unprecedented accuracy [16,17,18]. Furthermore, these technologies enable the precise detection of gait abnormalities that are frequently observed in individuals with neurological disorders [19,20]. For instance, Parkinson’s disease often manifests as a shuffling gait with reduced stride length, diminished arm swing, and episodic freezing, which reflects basal ganglia dysfunction [21,22]. Multiple sclerosis is typically associated with increased variability in step length and speed, as well as balance impairments due to demyelination affecting motor pathways [23,24]. Stroke survivors commonly exhibit hemiparetic gait, characterized by asymmetrical step lengths and decreased propulsion on the affected side, while cerebellar ataxia produces a wide-based, unsteady gait with irregular timing and coordination [25,26]. Huntington’s disease may lead to choreatic, unpredictable movements during walking [27]. These distinct patterns not only aid in early diagnosis but also guide the development of targeted rehabilitative strategies.

Data derived from human motion, particularly detailed gait measurements, offer valuable insights into the mechanisms underlying locomotion and the disruptions caused by neurological conditions [28,29]. Disruptions at any level can lead to significant alterations in kinematics, kinetics, and spatiotemporal gait parameters. Wearable sensors and advanced imaging tools capture complex datasets that provide detailed insights into joint angles, forces, and timing [30,31]. To manage and interpret these high-dimensional, non-linear data, sophisticated computational models such as support vector machines (SVMs), random forests (RFs), and neural networks (NNs) [32] are employed. Several studies have underscored the effectiveness of these AI-driven approaches in quantifying subtle motor impairments associated with neurological conditions, thereby enhancing clinical diagnostics and informing rehabilitative interventions [33,34,35].

To better understand the role of AI in diagnosing neurological diseases through biomechanical data and gait analysis, this study employed a bibliometric analysis of the existing literature. Bibliometric analysis is a well-established scientific method that provides a comprehensive overview of a field by identifying influential publications, key researchers, prominent institutions, and emerging research trends. A review of the literature revealed that bibliometric techniques have been widely applied to explore various topics related to neurological disorders [36,37,38,39,40,41,42]. Previous studies have employed these methods to examine the trends and advancements in decoding the applications of DL in neuroscience, as well as the broader use of AI in this field [43,44]. Similarly, bibliometric analyses have investigated the contributions of AI in neurosurgery [45]. Additionally, the role of biomechanics in stroke neurorehabilitation has also been explored through a bibliometric approach [46]. To date, no bibliometric study has integrated these three areas—AI, biomechanics, and neurological diseases—into a single, comprehensive analysis. Therefore, further research is required to not only clarify the intersection of these fields, but also to highlight existing research gaps to provide a foundation for further investigations.

These gaps are particularly significant given that (a) neurological diseases are increasingly diverse and prevalent; (b) AI represents a rapidly evolving field with immense potential; and (c) advancements in biomechanics provide valuable data for understanding and diagnosing these conditions. Therefore, the present study aims to examine the existing international literature through a bibliometric analysis and to review key topics identified using innovative bibliometric techniques to provide researchers and clinicians with a detailed understanding of the current state of AI applications in neurology, laying a strong foundation for future research and innovation.

## 2. Materials and Methods

### 2.1. Data Sources and Search Methods

**Figure 1:**
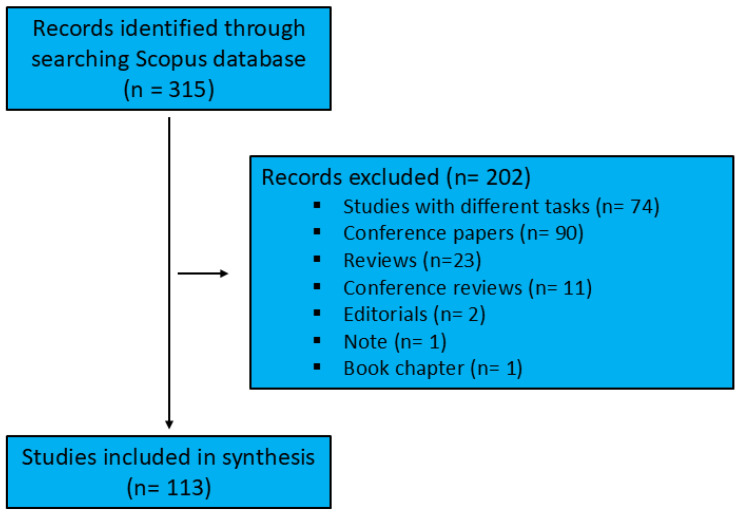
Literature search and selection workflow.

The documents analyzed in this study were sourced from the Scopus database (https://www.scopus.com/) on 16 January 2025. Scopus, recognized as one of the largest curated repositories of abstracts and citations spanning multiple disciplines [47], is an invaluable resource for bibliometric data analysis [48]. The advanced search function was utilized with a search string comprising compound keywords, structured using the BOOLEAN expression: (“neurological diseases” OR parkinson* OR alzheimer* OR epilepsy OR “epileptic seizures” OR stroke OR dementia OR “idiopathic tremor” OR “multiple sclerosis”) AND (“machine learning” OR “deep learning” OR “artificial intelligence”) AND (diagnosis OR detection OR diagnos*) AND (“biomechanical data” OR biomechanics OR “gait analysis”) between 1 January 2018 and 31 December 2024. Documents were included if these terms appeared in their titles, abstracts, or keywords. The initial search yielded 315 documents. Only original, English-language articles were included, excluding reviews (or other types of articles such as opinion papers) as well as articles published in conferences, books, or other non-academic journal outlets. Accepted studies were required to specifically focus on the application of artificial intelligence in diagnosing neurological diseases. The first two authors systematically reviewed the titles and abstracts and reached a consensus on their eligibility. After reviewing the 315 records, 202 were excluded as they did not address the inclusion criteria (Figure 1).

### 2.2. Data Analysis

The CSV file exported from Scopus was processed using VOSviewer (version 1.6.20) to perform a bibliometric analysis. VOSviewer, a free software tool, facilitates the creation, visualization, and exploration of bibliometric networks, making it particularly effective for mapping the trends and thematic structures of scientific fields [49]. Additionally, after converting the file into an Excel (xls) format using Microsoft Excel (Office 365, Microsoft Corporation, Redmond, WA, USA Excel 2024), the data were imported into Microsoft Power BI (Office 365, Microsoft Corporation, Redmond, WA, USA, Power BI Desktop 2025) for visualizing the distribution of documents over time. The bibliometric analysis included both performance analysis and scientific mapping, with the latter involving clustering techniques as part of the methodology [50,51,52].

### 2.3. Performance Analysis

The performance analysis included the following: (a) determining the annual number of published documents, (b) ranking the authors based on their citation count, and (c) ranking the sources with the highest number of publications.

### 2.4. Scientific Mapping

The scientific mapping techniques applied in this study included the following:

(a) Co-authorship analysis: Authors, organizations, and countries were analyzed as the unit of analysis, focusing on collaborations based on their co-authored documents. The analysis using authors as the unit of analysis was limited to those with at least two documents.
(b) Bibliographic coupling: Sources were examined as the unit of study to assess the extent to which two or more sources shared common references.
(c) Co-citation analysis: Sources were used as the unit of analysis to determine how frequently two or more sources were cited together in other documents. The analysis was limited to sources with at least ten citations.
(d) Co-occurrence analysis: The unit of analysis was “author keywords”, investigating how often two or more keywords appeared together within the same documents. “Author keywords” were limited to terms explicitly listed by the author as “keywords”. The minimum number of occurrences was set at two.

## 3. Results

### 3.1. Performance Analysis

**Figure 2:**
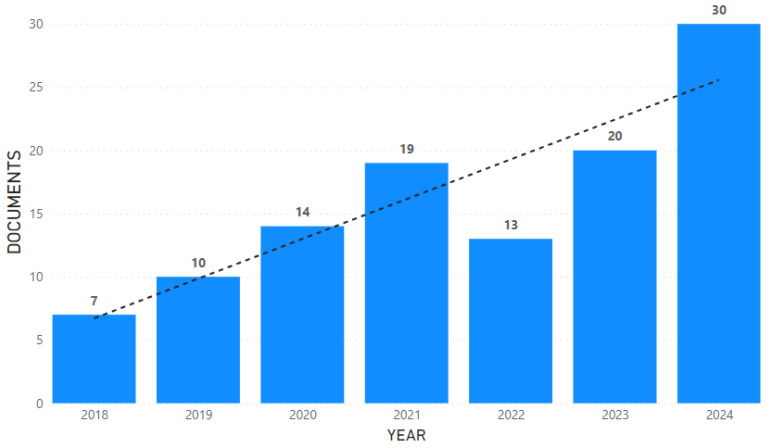
Annual publication trends (2018–2024). The bar chart shows the number of documents published each year from 2018 through 2024, with a dotted line indicating the overall trend.

A total of 113 articles were considered suitable for inclusion in our analysis. The bar chart (Figure 2) shows the annual number of documents published between the years 2018 and 2024. A noticeable upward trend can be observed, as depicted by the dotted trend line. There has been a steady increase in the number of documents published over the years, starting from seven in 2018 and peaking at thirty in 2024, despite a slight dip observed in 2022.

The key contributors and their significant impact within the field are presented in Table 1 below, showing a list of authors with a minimum of 100 citations, along with the number of documents they have contributed to. The authors Nöth, Orozco-Arroyave, and Vasquez-Correa lead the rankings with 227 citations each across two documents. Other notable contributors include Abdulhay, Arunkumar, Narasimhan, Vellaipapan, and Venkatraman, all with 203 citations and a single document. Klucken has a slightly lower citation count (193) with two documents.

Academic sources with a minimum of two published documents and their corresponding citation counts are listed in Table 2. The journal *Sensors* stands out as the leading source, with 16 documents and 373 citations, demonstrating its significant contribution to the field. *IEEE Transactions on Neural Systems and Rehabilitation Engineering* follows with five documents and 62 citations. Other notable sources include the *IEEE Journal of Biomedical and Health Informatics*, which has four documents and a substantial 249 citations, and *Expert Systems*, which has three documents and 201 citations.

### 3.2. Science Mapping

**Figure 3:**
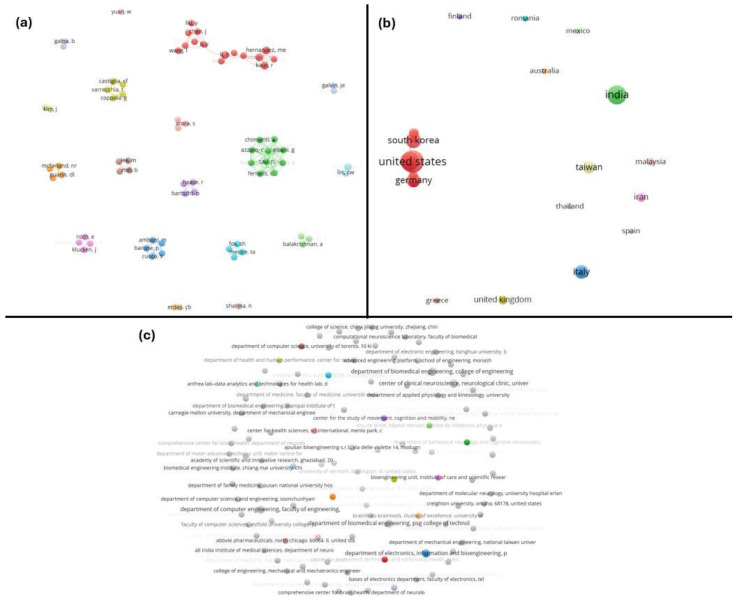
Co-authorship networks at multiple levels. Visualization of the collaboration networks in the field, analyzed at three levels: (**a**) authors, (**b**) countries, and (**c**) organizations. In each network, the nodes represent individual authors, countries, or institutions, and the edges indicate co-authored publications. Clusters of nodes (different colors) show groups that collaborate frequently.

The co-authorship networks presented in Figure 3 were analyzed at three levels: (a) authors, (b) countries, and (c) organizations. In panel (a), each node represents an individual author, while the connecting lines indicate co-authorship relationships. The visualization reveals that while some authors are central within their respective clusters, the sparse inter-cluster connections suggest limited collaboration between research groups in this field. In panel (b), countries are the unit of analysis. The United States, South Korea, and Germany emerge as key contributors, serving as central hubs within their networks. However, the overall lack of dense interconnections among countries indicates a significant gap in international collaboration, suggesting that greater global cooperation could enhance research outcomes. Panel (c) illustrates organizational-level collaborations, where nodes represent institutions. This panel similarly reveals minimal interaction between organizations, with most collaborations being localized and isolated. This observation highlights the need to foster stronger institutional partnerships to promote interdisciplinary and cross-institutional research in AI-driven neurological diagnostics.

**Figure 4:**
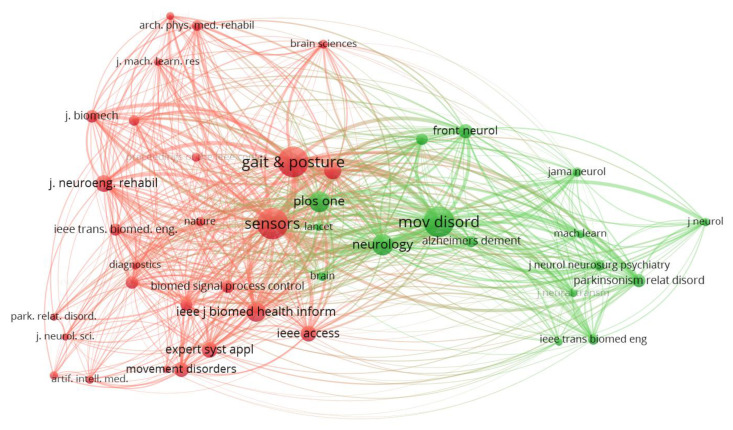
Co-citation analysis of sources. A network graph depicting how scientific sources (journals) are co-cited in the literature. The nodes represent journals (scaled by number of citations), and the edges connect journals that are often cited together, with thicker lines for stronger co-citation relationships. Two main color-coded clusters are evident: the red cluster groups journals (e.g., *Sensors*, *Gait & Posture*) that concentrate on sensor technologies and gait analysis, while the green cluster includes journals (e.g., *Movement Disorders*, *Neurology*) focused on neurological research and disorders (especially Parkinson’s disease).

A co-citation analysis of sources in Figure 4 shows the relationship between journals based on the frequency with which they are cited together in other documents. Each node represents a journal, while the size of the node reflects the number of times it has been cited. The connections (edges) between nodes indicate the strength of the co-citation relationship, with thicker lines representing stronger associations. The network is divided into distinct clusters, represented by different colors, which highlight the thematic groupings of journals. For instance, the red cluster includes journals such as *Sensors* and *Gait & Posture*, which are heavily focused on sensor-based systems and gait analysis. The green cluster, on the other hand, revolves around journals like *Movement Disorders* and *Neurology*, which are more aligned with neurological research and disorders such as Parkinson’s disease.

**Figure 5:**
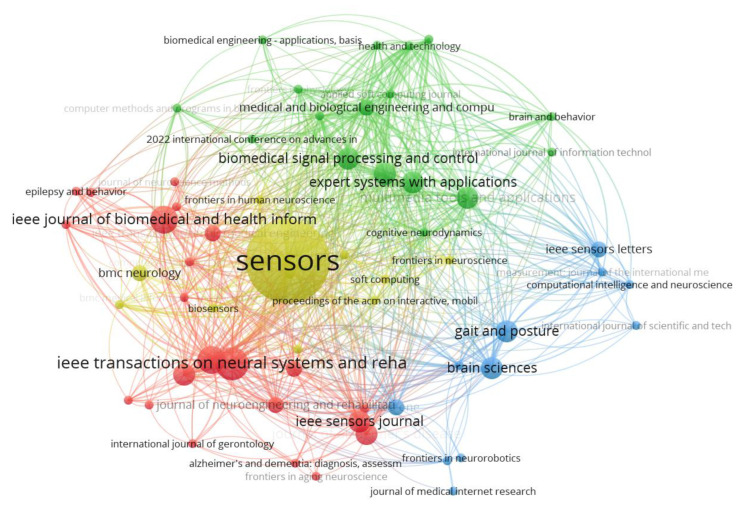
Bibliographic coupling network of sources. Network visualization of journals based on bibliographic coupling (shared references). Each node is a journal (with node size reflecting its number of publications or influence), and the edges link journals that cite similar references, forming thematic clusters. Four prominent clusters are labeled by color: the yellow cluster (dominated by the journal *Sensors*) is the most central, highlighting the strong interconnections in sensor technology and biomedical signal processing journals. The red cluster features journals like *IEEE J. Biomedical Health Informatics* and *IEEE Trans. Neural Systems & Rehab Engineering*, focusing on health informatics and neuroengineering. The green cluster is centered on interdisciplinary applied journals (e.g., *Expert Systems* with *Applications*), emphasizing computational approaches to biomedical problems. The blue cluster includes specialized journals such as *Gait & Posture* and *Brain Sciences*, reflecting a focus on movement analysis and neuroscience.

The bibliographic coupling network of journals in Figure 5 shows that each node represents a journal and the edges (connections) indicate the extent to which the journals share common references. The network is organized into distinct clusters, represented by different colors, highlighting thematic groupings based on shared references. The yellow cluster, dominated by *Sensors*, is the most central and influential, reflecting its extensive coupling with journals in fields like biomedical signal processing and sensor applications. The red cluster includes journals such as *IEEE Journal of Biomedical and Health Informatics* and *IEEE Transactions on Neural Systems and Rehabilitation Engineering*, which focus on health informatics and neural systems. The green cluster is centered around interdisciplinary journals like *Expert Systems* with *Applications and Biomedical Signal Processing and Control*, emphasizing computational and applied approaches. The blue cluster contains journals like *Gait & Posture* and *Brain Sciences*, which are more specialized in movement analysis and neuroscience.

**Figure 6:**
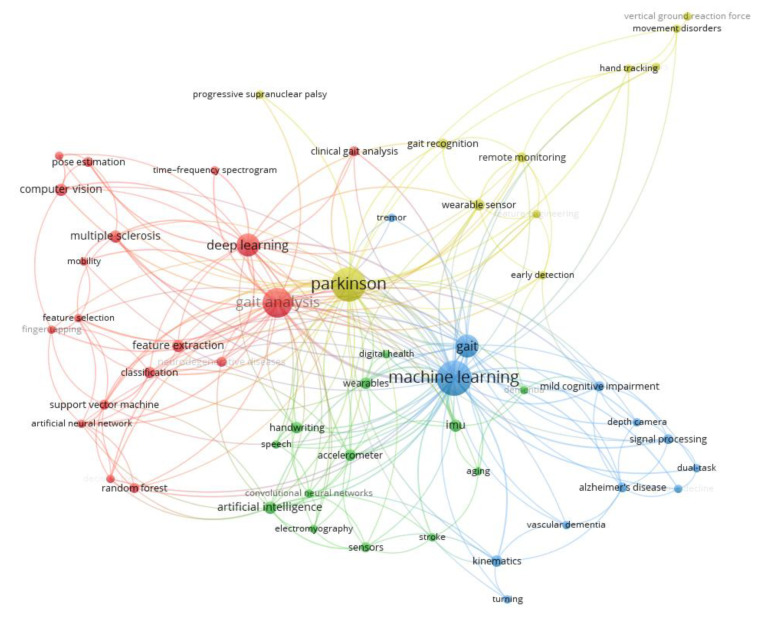
Keyword co-occurrence network (research themes). Network of author keywords illustrating four major thematic clusters in the literature. Each node represents a keyword (with its size proportional to its frequency of use), and the edges connect keywords that co-occur in the same articles, indicating a related topic association. Nodes are color-coded by cluster, corresponding to the distinct research themes identified in the field. Four clusters are highlighted (see also Table 3 for cluster details): (**a**) machine learning and gait analysis (e.g., keywords on gait parameters, classification algorithms), (**b**) sensors and wearable health technologies (keywords related to wearable devices, IMUs, remote monitoring), (**c**) cognitive disorders (terms regarding Alzheimer’s, MCI, cognitive testing with gait), and (**d**) neurological disorders and motion recognition (keywords on specific diseases like Parkinson’s, PSP, and motion capture methods). This figure encapsulates the core research themes, with closely connected keywords forming clusters that mirror the key topic areas driving the AI-based neurological diagnosis research.

The thematic structure of the research field based on a co-occurrence analysis of keywords is shown in Figure 6 and Table 3. The network in Figure 6 shows the relationships between keywords, with the nodes representing individual keywords and the edges indicating their co-occurrence in the same documents. The size of the nodes reflects the frequency of keyword usage, while the colors correspond to distinct clusters, representing different thematic areas. Table 3 complements the network by categorizing the clusters and listing the keywords associated with each thematic area. Four main clusters are identified (analyzed in detail in Section 4, the Discussion section).

Machine Learning and Gait Analysis (red cluster): This cluster includes keywords related to computational methods, such as “deep learning” and “support vector machine”, as well as applications in “gait analysis” and “pose estimation”. This theme highlights research focusing on the application of AI models to analyze gait patterns for the early diagnosis and monitoring of neurological diseases, such as Parkinson’s disease and multiple sclerosis. The practical significance lies in advancing non-invasive diagnostic methods that can identify motor dysfunctions at an early stage, enabling timely intervention and personalized care.

Sensors and Wearable Health Technologies (green cluster): Keywords in this cluster focus on sensor-based systems and wearable technologies, such as “accelerometer”, “IMU”, and “digital health”. This theme encompasses the development and use of wearable devices equipped with AI for continuous health monitoring. The significance of this cluster is in its potential to facilitate remote patient monitoring, reducing the need for hospital visits and enabling proactive health management.

Cognitive Disorders (blue cluster): This cluster encompasses terms related to neurological and cognitive impairments, including “Alzheimer’s disease”, “cognitive decline”, and “dual-task”. Studies in this cluster focus on using AI to analyze cognitive and motor functions to detect disorders such as Alzheimer’s disease and mild cognitive impairment. The practical application of this cluster is the enhancement of early diagnostic accuracy, allowing for timely interventions and better disease management.

Neurological Disorders and Motion Recognition Technologies (yellow cluster): Keywords in this cluster highlight technologies for the analysis and management of movement disorders, such as “Parkinson”, “gait recognition”, and “remote monitoring”. This cluster involves the application of AI-powered motion recognition tools to detect and monitor motor impairments, particularly in diseases such as Parkinson’s disease. The practical significance is the potential for continuous monitoring, improving rehabilitation strategies, and enhancing personalized treatment plans.

## 4. Discussion

### 4.1. Thematic Areas

Our bibliometric science mapping included a co-occurrence analysis of author keywords to identify major research themes. Using VOSviewer software, we built a keyword co-occurrence network in which each node represented a keyword and the edges reflected the frequency of two keywords appearing together in the same paper. We applied the association strength clustering algorithm (via VOSviewer) to detect groups of closely related keywords, which represented distinct thematic clusters in the field. To focus on significant and recurring topics, a minimum occurrence threshold was set—keywords had to appear in at least two publications to be included. This threshold filtered out single-use or rare terms that could create noise, ensuring that each identified cluster was built around a meaningful research topic. As a result of this analysis, four thematic clusters emerged (visualized in Figure 6 and detailed in Table 3), each corresponding to a prominent research theme in AI-driven neurological disease diagnosis. The co-occurrence clustering revealed four primary research themes. Each cluster highlighted a practical area of focus in which AI is being leveraged to advance neurological diagnosis.

#### 4.1.1. Machine Learning and Gait Analysis

The specific focus is on the development and application of ML techniques for analyzing and diagnosing neurological disorders using gait data, with a particular emphasis on conditions such as Parkinson’s disease, multiple sclerosis, and other neurodegenerative diseases. Common themes across these studies include the use of gait data and ML techniques to detect motor abnormalities, the enhancement of diagnosis through DL algorithms, and the high accuracy achieved in classifying patients from healthy controls. Specifically, these studies employ a variety of approaches for data collection and analysis, such as motion sensors, vGRF analysis, EMG, and 3D gait kinematics [53,54,55,56,57,58,59,60]. Furthermore, the included studies often employ ML, including techniques such as RFs, decision trees, SVMs, and DL to classify or assess neurological diseases based on gait characteristics.

The application of specialized tools and methods for detecting early motor changes is also a recurring theme in the studies, enabling early diagnosis. Additionally, the personalization of approaches is crucial for enhancing accuracy and improving clinical monitoring. The findings indicate that the combined use of these techniques can significantly improve diagnostic procedures and support the monitoring of therapeutic outcomes in patients [54,55,61,62,63]. Ultimately, these studies highlight the versatility of modern ML algorithms and data analysis methods in identifying motor disorders, offering new opportunities for the more accurate and earlier diagnosis of neurodegenerative diseases.

Overall, these studies demonstrate the importance of combining gait analysis with ML and DL techniques for the accurate diagnosis and monitoring of neurological and non-neurological disorders. These technologies not only provide new possibilities for early diagnosis and patient monitoring, but also allow for a deeper understanding of the motor problems associated with various diseases. Looking ahead, advancements in AI-powered gait analysis hold the potential to revolutionize personalized healthcare.

#### 4.1.2. Sensors and Wearable Health Technologies

AI-powered sensors and wearable devices are revolutionizing health monitoring, enabling real-time data analysis for applications ranging from handwriting, movement analysis, and speech tracking to stroke recovery and digital health management. Utilizing technologies such as inertial measurement units (IMU) and sensors can enable the creation of personalized, continuous monitoring systems that process large-scale data efficiently [64,65,66,67,68]. The integration of AI into wearable devices represents a significant step forward in preventive care and long-term health management.

A common thread across these studies is the need for enhanced accuracy in AI algorithms, particularly in applications for elderly patients and those with neurological conditions such as Parkinson’s [69,70,71,72]. Another critical focus is developing devices that can seamlessly and effectively integrate into patients’ daily lives, promoting independence and improving quality of life. Although significant progress has been made in detecting motor abnormalities, these systems must be fine-tuned for widespread clinical use.

In conclusion, wearable technologies powered by AI are aimed at transforming healthcare by offering new possibilities for the early diagnosis and continuous monitoring of diseases such as Parkinson’s disease. As research advances, these innovations will enable clinicians to provide more personalized, effective treatments while improving patient outcomes and quality of life.

#### 4.1.3. Cognitive Disorders

The use of AI to understand and diagnose cognitive disorders is a vital focus in this cluster. Researchers are particularly interested in age-related neurological conditions, such as Alzheimer’s disease, mild cognitive impairment (MCI), and vascular dementia. AI is being employed to analyze data from tools like depth cameras and sensors, which help to assess behavior, cognition, and motor abilities. Techniques such as AI-driven dual-task performance analysis and tremor pattern detection have shown promise in identifying early signs of cognitive decline [73,74,75,76,77,78,79,80,81,82]. This study highlights the potential of AI to revolutionize early diagnosis, clinical monitoring, and intervention strategies for cognitive disorders.

A common thread across these studies is the integration of AI, ML, and motion analysis with other behavioral data to enable comprehensive assessments of cognitive health. The combination of these technologies may allow researchers to provide an early and a more accurate diagnosis, enabling timely intervention and better management of cognitive disorders. This holistic approach has the potential to significantly improve the quality of life for patients and reduce the burden on caregivers.

Overall, this cluster underscores the importance of leveraging AI to address the growing challenges of cognitive decline. By offering tools for early diagnosis and personalized care, these innovations open new pathways for clinical intervention to improve cognitive health outcomes.

#### 4.1.4. Neurological Disorders and Motion Recognition Technologies

This thematic area integrates motion recognition technologies and AI to study neurological disorders like Parkinson’s disease, progressive supranuclear palsy, and other movement disorders. Researchers have leveraged motion analysis for early detection using tools such as remote monitoring, vertical ground reaction force data, and hand tracking. These technologies, often paired with wearable sensors powered by AI, allow for the continuous tracking of symptoms such as tremors and gait abnormalities. By improving remote monitoring systems, these studies pave the way for the scalable and efficient management of chronic neurological conditions.

Beyond Parkinson’s disease, the prediction of global cognitive function decline using ML offers a valuable tool for identifying risk in older adults. By analyzing gait and physical fitness parameters, researchers can enable earlier interventions that improve the quality of life for the elderly [83,84,85,86,87,88,89,90]. Additionally, combining dual-task gait evaluations with ML may provide more comprehensive cognitive assessments to enhance the diagnostic accuracy for mild cognitive impairment, as well as to create opportunities for early intervention.

A recurring theme has been identified across these studies, which is the integration of cutting-edge technologies, such as motion analysis and ML, to enable the precise and early diagnosis of neurological and cognitive decline. By continuing to develop and refine these tools, researchers are driving advancements in personalized care, offering hope for better disease management and improved patient outcomes.

#### 4.1.5. Qualitative Critique of AI Methodologies in Neurological Diagnosis

Model Explainability: A pervasive challenge in applying AI to neurological disease diagnosis is the explainability of models. Many AI systems, particularly deep learning networks, function as “black boxes”, making it difficult for clinicians to understand the basis of their predictions. This lack of transparency can hinder clinical trust and adoption. Explainable AI (XAI) frameworks are increasingly emphasized to address this issue. By providing interpretable insights (e.g., highlighting which gait features or biomarkers influenced a decision), XAI can enhance clinician and patient confidence in AI-driven diagnoses. It also helps to meet the regulatory requirements for transparency and safety in medical AI. Balancing high model performance with interpretability remains an active area of research, but it is crucial to integrate AI into routine neurological practice.

Need for External Validation: Another critical limitation is the lack of robust external validation for many AI models in neurology. Most studies have developed and tested models on the same dataset or within a single center, which constrains their generalizability to broader patient populations and clinical settings. External validation studies on medical AI are relatively rare and often reveal a drop in performance on new data. For example, in radiology AI, most algorithms show diminished accuracy on external datasets compared with their original development data. This underscores the importance of validating neurological AI diagnostic tools across diverse cohorts and settings. Prospective multi-center trials and independent test sets should be used to ensure that models truly generalize to different hospitals, scanning devices, or patient demographics. Strengthening external validation will improve the reliability and clinical utility of AI diagnostic systems.

Ethical Considerations: The deployment of AI for neurological diagnoses raises important ethical issues that must be addressed. Data privacy and security are foremost concerns, since AI models require large volumes of sensitive patient data (e.g., neuroimaging, gait sensor data) for training. Ensuring compliance with privacy regulations (such as GDPR and HIPAA) and using stringent data anonymization and encryption protocols are essential to protect patient confidentiality. Another concern is algorithmic bias and fairness. AI models trained on homogeneous or unrepresentative data may inadvertently perpetuate healthcare disparities. For instance, an algorithm developed mostly on data from one demographic group might perform poorly in others, leading to unequal diagnostic accuracy. Researchers must strive to use diverse training data and bias-mitigation techniques to ensure equity. Moreover, the accountability of AI decisions is an ethical consideration: clear guidelines are needed for how AI recommendations are used in clinical decisions and who is responsible in cases of error or misdiagnosis. Addressing these ethical challenges—privacy, bias, and accountability—will foster trust in AI systems and promote their safe integration into neurological healthcare.

#### 4.1.6. Underlying Causes, Implications, and Solutions for Research Gaps

Underlying Causes of Research Gaps: The research gaps identified in this study stem from multiple underlying causes. Limited interdisciplinary communication, restricted access to shared datasets, and a fragmented approach to AI development in neurology have contributed to these gaps. Furthermore, many studies rely on single-center data, limiting diversity and external validation. The lack of standardized protocols for data collection and algorithm validation further exacerbates these challenges.

Implications for Research and Practice: These gaps have significant implications. Biased or under-validated AI models may result in a limited clinical applicability, reducing their effectiveness in diverse real-world settings. Additionally, the absence of international collaborations restricts the sharing of diverse data and insights, potentially slowing innovation and limiting the global applicability of research findings. Addressing these gaps is essential to improve diagnostic accuracy, ensure equity in care, and advance AI innovation in neurology.

Potential Solutions: To mitigate these challenges, several actionable solutions are proposed. Firstly, fostering interdisciplinary and international collaborations is essential for sharing diverse perspectives and resources. Secondly, encouraging the development and use of multi-center datasets will enhance model robustness and generalizability. Thirdly, adopting explainable AI (XAI) frameworks will increase trust in AI models and promote their clinical acceptance. Finally, promoting the development of standardized protocols for AI validation and regulatory oversight will ensure ethical, reliable, and practical AI applications in neurology.

### 4.2. Publication Trends and Patterns

Our study conducted a comprehensive bibliometric analysis to investigate research trends in the field of AI for the diagnosis of neurological diseases using biomechanical data from the current literature. The overall trend shows a consistent and steady growth from 2018 to 2024, with a slight dip observed in 2022, which confirms the increasing interest and activity of research in this field over time.

The journals listed in Table 2 cover a wide range of scientific fields, reflecting the interdisciplinary nature of the subject. Specifically, they represent areas such as electronics and computational engineering in journals such as *IEEE Transactions on Neural Systems and Rehabilitation Engineering*, *IEEE Journal of Biomedical and Health Informatics*, and *IEEE Sensors Journal*, which focus on the use of technology and computational systems for health research and applications. In addition, journals belonging to the field of medicine, biomedical science, and neuroscience, such as *Sensors*, *Biomedical Signal Processing and Control*, *Journal of Alzheimer’s Disease*, *Journal of Neuroengineering and Rehabilitation*, and *Parkinsonism and Related Disorders*, have also been included. Collectively, these journals emphasize the multifaceted study and technological advancement of diagnostic, monitoring, and rehabilitation approaches for human health systems and neurological disorders. The inclusion of journals like *Expert Systems and Multimedia Tools and Applications* highlights the growing role of intelligent systems and data analytics in modeling and addressing complex health-related problems. These multifaceted advancements highlight the importance of interdisciplinary collaboration across science, technology, and medicine to address intricate research challenges.

The analysis of both the bibliographic coupling network and the co-citation network of sources provides important insight into the structure and dynamics of the research field. The bibliographic coupling network highlights a high level of thematic cohesion among journals, with strong bibliographic ties indicating interconnectedness within the field. At the same time, the presence of distinct clusters points to the diversity of subfields, ranging from sensor technologies to movement analysis and computational applications. On the other hand, the co-citation network reveals that, while there is strong interconnectedness within individual clusters, cross-cluster interactions remain relatively limited. This thematic segmentation underscores the need for interdisciplinary approaches to bridge the gaps between sensor-based technologies and neurological research. Addressing this fragmentation could lead to a more integrated understanding and foster innovation by connecting related but currently isolated areas of research to breach the gap.

Another very important finding of the present study is the lack of international collaboration. The limited connectivity at all three levels (authors, countries, and organizations) of the co-authorship analyses underscores the fragmented nature of collaborative efforts in the studied field, indicating potential opportunities to foster greater cooperation and network development. Collaboration and networking in scientific research play a crucial role in enhancing productivity and scientific progress [50,51,52]. Strengthening global partnerships and developing cross-institutional networks could lead to more comprehensive and impactful research outcomes in the field.

Our study identified several critical research gaps in the application of AI in the diagnosis of neurological diseases. One major limitation is the lack of robust external validation for AI models, which constrains their generalization with diverse populations and clinical settings. Furthermore, the explainability and interpretability of AI systems, particularly deep learning models, remain significantly challenging. Often functioning as “black boxes”, these systems make it difficult for clinicians to understand or trust their predictions, thus emphasizing the need for explainable AI frameworks that enhance clinical acceptance. Another notable issue is the reliance on small or homogeneous datasets, which fail to capture the variability present in broader patient populations. Larger, multi-center datasets are essential to ensure the reliability and applicability of AI models across different settings. Additionally, the thematic segmentation observed in bibliometric networks highlights the fragmented nature of research efforts, underscoring the importance of interdisciplinary collaboration among experts in AI, biomechanics, and neuroscience. Enhanced collaboration across these fields could drive innovation by integrating complementary expertise. Lastly, there is a pressing need for studies that assess the performance of AI systems across diverse populations, including variations in age, ethnicity, and socioeconomic backgrounds. Addressing these gaps will not only improve the accuracy and reliability of AI diagnostic tools but also promote equity and inclusivity in their application. Coordinated efforts from interdisciplinary research teams will leverage advanced computational techniques and foster collaboration across institutions and countries. By overcoming these limitations, the field can move towards more accurate, explainable, and widely applicable AI systems for the diagnosis and management of neurological disorders.

Overall, the increasing research focus on AI-driven gait analysis has direct implications for early diagnosis and remote monitoring in clinical practice. AI-based diagnostic tools could also influence healthcare policy by optimizing patient triage, enhancing resource allocation, and improving access to specialized care. Recognizing these connections underscores the transformative potential of AI in enhancing patient outcomes and healthcare efficiency.

### 4.3. Limitations

Although valuable insights have been identified in research trends and thematic areas in the field of AI for the diagnosis of neurological diseases using biomechanical data, the present study has certain limitations: (1) the analysis was based solely on data sourced from the Scopus database, which, while comprehensive, may exclude relevant articles indexed in other databases such as PubMed or Web of Science; (2) the focus on English-language articles might have led to the exclusion of significant contributions published in other languages; (3) the exclusion of articles from conferences and books might have overlooked some important contributions to the field that have not yet been published in academic journals; (4) the bibliometric approach inherently emphasizes quantitative metrics, which may not fully capture the qualitative impact of specific studies; although, a brief qualitative synthesis of the four main thematic areas was conducted in our study; and (5) despite the fact that the search terms employed in this study generally covered neurological diseases and some common conditions, they did not encompass several specific neurological conditions—including migraines (where gait problems are typically not expected), hydrocephalus, spinal disease, motor neuron disease, neuropathy, myasthenia gravis, and myopathies. To sum up, future research should consider integrating multi-database searches and expanding the inclusion of diverse languages to achieve a more comprehensive overview of this field.

## 5. Conclusions

The present study provides a strong foundation for the development of robust and effective AI-driven healthcare solutions, which is crucial for future research and for the need for interdisciplinary collaboration among researchers. The bibliometric analysis focuses specifically on the application of AI in diagnosing and managing neurological disorders related to biomechanics and gait analysis, bridging the gap of current trends and research patterns. The analysis of 113 studies published between 2018 and 2024 identified key thematic areas—machine learning and gait analysis, sensors and wearable health technologies, cognitive disorders related to movement impairments, and motion recognition technologies—which reflect the diverse and interdisciplinary nature of AI applications in neurology, particularly within the scope of biomechanics. The analysis revealed a clear trend of increasing research interest over the years, underscoring the growing recognition of AI’s transformative potential in neurological disorders associated with movement and motor function. Furthermore, the present findings highlight how the convergence of disciplines such as neurology, data science, and occupational therapy can foster collaboration to address complex motor-related neurological conditions. By focusing on the intersection of biomechanics and AI, this study contributes to a more specialized understanding of the role of AI in neurological healthcare applications.

To advance the integration of AI technologies into clinical applications for neurological disease diagnosis, future research should prioritize several key areas. First, interdisciplinary collaboration is essential to bring together AI experts, neurologists, data scientists, and healthcare policymakers to ensure the development of clinically relevant and ethically sound AI tools. Establishing standardized datasets, including diverse demographic and clinical data, will improve the generalizability and robustness of the AI models. Moreover, emphasis should be placed on developing explainable AI (XAI) frameworks that enhance transparency and trust among clinicians and patients. Real-world clinical validation, through multi-center trials and diverse population testing, is crucial for transitioning AI models from research to practice. Finally, healthcare systems and policymakers must collaboratively establish regulatory guidelines and frameworks that facilitate the safe and effective adoption of AI in clinical settings. Addressing these areas will bridge the gap between AI technology and its practical application, ultimately improving diagnostic accuracy, patient outcomes, and healthcare delivery.

## Figures and Tables

**Table 1:** Top contributing authors with over 100 citations and their document counts.

Author	Citations	Documents
Nöth, E	227	2
Orozco-Arroyave, Jr	227	2
Vasquez-Correa, Jc	227	2
Abdulhay, E	203	1
Arunkumar, N	203	1
Narasimhan, K	203	1
Vellaiappan, E	203	1
Venkatraman, V	203	1
Klucken, J	193	2
Bilodeau, Ga	189	1
Bouachir, W	189	1
El Maachi, I	189	1
Arias-Vergara, T	169	1
Eskofier, B	169	1
Fox, Sh	113	2
Li, Mh	113	2
Mestre, Ta	113	2
Taati, B	113	2

**Table 2:** Key academic sources with a minimum of two documents and their citation impact.

Source	Documents	Citations
*Sensors*	16	373
*IEEE Transactions on Neural Systems and Rehabilitation Engineering*	5	62
*IEEE Journal of Biomedical and Health Informatics*	4	249
*Frontiers in Neurology*	4	35
*Expert Systems*	3	201
*Biomedical Signal Processing and Control*	3	73
*Gait & Posture*	3	59
*Journal of Alzheimer’s Disease*	3	56
*IEEE Access*	3	43
*Brain Sciences*	3	35
*Parkinsonism and Related Disorders*	3	33
*IEEE Sensors Journal*	3	18
*Multimedia Tools and Applications*	3	13
*Journal of Neuroengineering and Rehabilitation*	2	98
*Plos One*	2	51
*IEEE Sensors Letters*	2	45
*BMC Neurology*	2	27
*IEEE Transactions on Biomedical Engineering*	2	27
*Medical and Biological Engineering and Computing*	2	12
*International Journal of Advanced Computer Science and Applications*	2	8

**Table 3:** Clustered keywords representing key research themes.

Machine Learning and Gait Analysis (red cluster)	artificial neural network, classification, clinical gait analysis, computer vision, decision tree, deep learning, feature extraction, feature selection, finger tapping, gait analysis, levodopa-induced dyskinesia, mobility, multiple sclerosis, neurodegenerative diseases, pose estimation, random forest, support vector machine, time-frequency spectrum
Sensors and Wearable Health Technologies (green cluster)	accelerometer, aging, artificial intelligence, convolutional neural network, dementia, digital health, electromyography, handwriting, IMU, sensors, speech, stroke, wearables
Cognitive Disorders (blue cluster)	Alzheimer’s disease, cognitive decline, depth camera, dual-task, gait, kinematics, machine learning, mild cognitive impairment, signal processing, tremor, turning, vascular dementia
Neurological Disorders and Motion Recognition Technologies (yellow cluster)	early detection, feature engineering, gait recognition, hand tracking, movement disorders, Parkinson, progressive supranuclear palsy, remote monitoring, UPDRS, vertical ground reaction, wearable sensor
